# Air Pollution Exposure Assessment for Epidemiologic Studies of Pregnant Women and Children: Lessons Learned from the Centers for Children’s Environmental Health and Disease Prevention Research

**DOI:** 10.1289/ehp.7673

**Published:** 2005-06-24

**Authors:** Frank Gilliland, Ed Avol, Patrick Kinney, Michael Jerrett, Timothy Dvonch, Frederick Lurmann, Timothy Buckley, Patrick Breysse, Gerald Keeler, Tracy de Villiers, Rob McConnell

**Affiliations:** 1 Department of Preventive Medicine, Keck School of Medicine, University of Southern California, Los Angeles, California, USA; 2 Mailman School of Public Health, Columbia University, New York, New York, USA; 3 School of Public Health, University of Michigan, Ann Arbor, Michigan, USA; 4 Sonoma Technology, Inc., Petaluma, California, USA; 5 Bloomberg School of Public Health, Johns Hopkins University, Baltimore, Maryland, USA

**Keywords:** air pollution, airborne, ambient, Centers for Children’s Environmental Health and Disease Prevention Research, Children’s Centers, cohort study, direct measurement, exposure assessment, modeling, National Children’s Study, personal measurement

## Abstract

The National Children’s Study is considering a wide spectrum of airborne pollutants that are hypothesized to potentially influence pregnancy outcomes, neurodevelopment, asthma, atopy, immune development, obesity, and pubertal development. In this article we summarize six applicable exposure assessment lessons learned from the Centers for Children’s Environmental Health and Disease Prevention Research that may enhance the National Children’s Study: *a*) Selecting individual study subjects with a wide range of pollution exposure profiles maximizes spatial-scale exposure contrasts for key pollutants of study interest. *b*) In studies with large sample sizes, long duration, and diverse outcomes and exposures, exposure assessment efforts should rely on modeling to provide estimates for the entire cohort, supported by subject-derived questionnaire data. *c*) Assessment of some exposures of interest requires individual measurements of exposures using snapshots of personal and microenvironmental exposures over short periods and/or in selected microenvironments. *d*) Understanding issues of spatial–temporal correlations of air pollutants, the surrogacy of specific pollutants for components of the complex mixture, and the exposure misclassification inherent in exposure estimates is critical in analysis and interpretation. *e*) “Usual” temporal, spatial, and physical patterns of activity can be used as modifiers of the exposure/outcome relationships. *f*) Biomarkers of exposure are useful for evaluation of specific exposures that have multiple routes of exposure. If these lessons are applied, the National Children’s Study offers a unique opportunity to assess the adverse effects of air pollution on interrelated health outcomes during the critical early life period.

A major study design challenge for the National Children’s Study will be to maximize and characterize exposure contrasts in its cohort of 100,000 pregnant women residing in multiple locations across the United States, thereby enhancing the power to estimate exposure–response relationships from childhood into adulthood. Multiple outcomes are of interest, including pregnancy outcomes, neurodevelopment, asthma, obesity, and pubertal development. Exposures to a wide spectrum of environmental pollutants are being considered for investigation in the study, including air pollutants of indoor and outdoor origin (National Children’s Study 2004).

Given the pollutants and health endpoints currently under consideration, exposure assessment for the variable periods during pregnancy, infancy, and childhood will be needed. For asthma-related outcomes, daily, monthly, yearly, and multiyear exposure metrics with varying time integration periods may be required. For pregnancy outcomes, monthly estimates as well as estimates for critical periods may be needed. For neurodevelopment, monthly, yearly, and multiyear metrics may be most relevant. For these and other outcomes, time-integrated average levels may capture the effects of chronic exposure during specific periods, but more discrete and intense sampling frequency or duration may be needed to better assess specific exposure–response relationships.

The purpose of this article is to summarize exposure assessment lessons learned in the Centers for Children’s Environmental Health and Disease Prevention Research (hereafter Children’s Centers) for air pollutants and health outcomes of National Children’s Study interest. Exposures to allergens and bioaerosols are considered elsewhere in this mini-monograph. Many of the Children’s Centers have active research programs involving the assessment of air pollution in epidemiologic studies (Table 1). On the basis of experience of investigators from these centers, we provide recommendations for air pollution exposure assessment consideration in the study design, population selection, exposure data collection, analysis, and interpretation of findings of the National Children’s Study.

## Lessons Learned in Air Pollution Exposure Assessment

An essential design element of environmental epidemiologic studies is the *a priori* consideration of exposure assessment to ensure that the study exposure range will maximize the ability to evaluate key exposure–response relationships (Navidi et al. 1994, 1999). Study population selection and exposure assessment design are linked. Successful selections require consideration of the developmental time frames of interest and the biologic outcome mechanisms, in addition to understanding the spatial characteristics of airborne indoor and ambient exposures. One potentially successful design strategy is to maximize the number of contrasting pollution profiles among study subjects by using a quasi-factorial approach to select populations distributed over geographic regions with different pollution profiles (and/or including homes with different indoor sources and proximity to specific sources) (Gauderman et al. 2000).

The National Children’s Study proposes to investigate the relationships between patterns and histories of exposure during critical periods and the development of disease in later life. This creates an inherent tension because exposure assessment in large cohort studies requires a compromise between the optimal information obtained from individual measurements and feasibility constraints related to sampling methods, respondent burden, and cost. Feasibility considerations likely dictate that direct measurements will be limited to subsets of subjects monitored for short time periods (“snapshots”) in selected microenvironments, whereas exposure metrics used in chronic effects analyses for the entire cohort will be time-integrated over extended periods (days to months). The proposed size and duration of the National Children’s Study will require the use of modeling to estimate time-integrated exposures for the entire cohort even when direct measurements using snapshots of exposure are available for subsets of the cohort.

Several modeling frameworks are applicable to the National Children’s Study. Basic approaches rely on using questionnaire responses as a surrogate for exposure and on assigning exposures based on air pollutants measured at a central monitor. The latter approach has been successfully employed to detect significant health effects (Dockery et al. 1993; Gauderman et al. 2002; Pope et al. 2002; Ritz et al. 2000; Samet et al. 2000). More refined approaches allow for estimation within communities using dispersion models and information on transport, land use, and meteorology (Brauer et al. 2002; English et al. 1999; Finkelstein et al. 2003; Hoek et al. 2002; Nafstad et al. 2004). Considerations for modeled exposures include the availability of high-quality input data on the appropriate geographic scale and the need for validation and calibration studies to enable exposure uncertainty assignments. There are important limitations of modeling air pollution exposures (Sarnat et al. 2001). Studies indicate that for some pollutants, such as particulate matter (PM) and volatile organic compounds, indoor sources can predominate (Sax et al. 2004; Tonne et al. 2004; Wallace et al. 2004). Any strategy that relies on ambient modeling should also attempt to assess indoor exposures in subsamples of homes and thorough questionnaire or inspection data that examine important potential sources such as smoking habits or the presence of an attached garage. This is especially needed for air pollutants for which indoor sources are often the most significant contributors (Payne-Sturges et al. 2004). Understanding and assessing the role of exposure measurement error in health effects assessment are central issues for the design and implementation of health effect cohort studies (Jerrett and Finkelstein 2005).

Finally, interpretation of National Children’s Study findings will require information about specific pollutant surrogates because of the complex mixture of covarying pollutants in respirable air (Manchester-Neesvig et al. 2003). Pollutants covary because they are emitted from common sources or are produced by common atmospheric chemistry and meteorologic processes. Identification of source contributions within specific geographic regions may enhance interpretability of single pollutant associations with health outcomes (Laden et al. 2000; Samet et al. 2000).

In the following sections, we provide recommendations and issues that may need to be considered in implementing them. These are supported by some specific examples from the Children’s Centers listed in Table 1.

## Specific Recommendations

### National Children’s Study subject selection.

Study populations should be selected to maximize spatial exposure contrasts for the pollutants of interest. Because multiple pollutants are of interest for the National Children’s Study, priorities must be established to allow identification of individuals with a wide range of exposure profiles for those key pollutants of study interest.

Issues to consider include spatial scale variations of pollutants, in order to select a study population that maximizes exposure contrasts (Table 2). Table 2 identifies the spatial scales of variability for ambient pollutants to consider in the study design for the National Children’s Study. The scales are categorized as regional (100–1,000 km), urban (4–50 km), neighborhood (50 m to 4 km), and household (≤50 m, including outdoor and indoor microenvironments). For some exposures, contrast in exposure can be achieved by considering indoor sources and behavior (e.g., smoking vs. nonsmoking homes), if indoor-source pollutant health effects are of interest. For PM, the spatial scale variability of importance depends on the constituents of interest. For example, elemental carbon (EC) from ambient primary combustion processes varies on urban and neighborhood scales. Indoor sources from combustion also contribute to personal EC exposure (LaRosa et al. 2002). In contrast, particulate sulfates typically vary on a regional scale. To maximize exposure gradients to EC, subjects would need to be selected on a neighborhood scale, such as based on distance to busy roadways. Sulfates’ regional nature would be better reflected in a subject selection scheme involving different regions of the United States.

To select subjects based on exposure contrasts for ambient pollutants (e.g., ozone, sulfate), exposure data on geographic variation in levels and spatial gradients over time are needed. For criteria pollutants, existing data are available from a national network of monitoring stations. Data for many other pollutants of biologic interest may be sparse or nonexistent (e.g., EC and air toxics). In addition, for other pollutants with both indoor and outdoor sources (e.g., PM mass, nitrogen oxides, volatile organic compounds), much of the variability in exposure is driven by indoor source activity and/or very proximate local sources (e.g., traffic). For these pollutants, levels may need to be measured or modeled with the appropriate spatial and temporal resolution in pilot studies to ascertain the appropriate spatial, temporal, and behavioral determinants. In addition to variable pollutant source strengths, subject-specific temporal–spatial–physical patterns of activity may meaningfully affect both within and between-group exposure assignments. Capturing this variability in applicably useful ways for large study population studies is challenging and often a multi-faceted approach using self-administered questionnaires, walk-through surveys, instrument deployments, and sentinel monitoring.

Because several pollutants of interest for the National Children’s Study are regional in nature, subject selection from areas with contrasting pollution profiles is likely to be most informative. The national scope of the National Children’s Study provides the opportunity to maximize the number of study profiles. For example, the constituents of PM <2.5 μm in diameter (PM_2.5_) within a region are highly correlated, but between regions the correlations may be lower. PM_2.5_ sulfate is higher in the eastern United States and lower in the western United States, whereas PM_2.5_ nitrate is lower in the eastern United States and higher in the western United States. Therefore, the comparable effect of these PM_2.5_ constituents may be separable by study design. Replication of pollution profiles in different regions is also important to allow for effects of geographic variables such as weather and other confounding variables to be controlled in the analyses (Jerrett et al. 2003a, 2003b; Krewski et al. 2000; Peters 1997; Peters et al. 1999a). Exposures within homes with common sources are also highly correlated and may be separated by design.

An example of the integration of these approaches is the Southern California Children’s Health Study (CHS), a study performed by investigators in the University of Southern California (USC)/University of California at Los Angeles Children’s Environmental Health Center. The USC CHS is a multiyear cohort study of several thousand southern California school children (Berhane et al. 2004; Kunzli et al. 2003; Peters 1997). The primary USC CHS research question is whether ambient air pollution causes chronic adverse respiratory health effects during childhood and adolescent growth and development. Almost 12,000 children from schools in 13 southern California communities have been recruited into five cohorts since the study began in 1993.

Communities were selected to maximize differences in outdoor air pollutant concentrations. To distinguish the effects of different pollutants, communities were selected to minimize the spatial correlations between three priority study pollutants [O_3_, nitrogen dioxide, and PM < 10 μm in diameter (PM_10_)]. However, the full quasi-factorial design could not be fulfilled because all the potential pollution profiles do not occur in nature. Specific community selections were based on historical air pollution levels for several years before study inception, exposure patterns, and census demographic data. Because of differences in the number of locations at which pollutants were measured and the frequency and type of measurements made, data available for selecting communities were more reliable for O_3_ than for PM_10_, and more reliable for PM_10_ than for NO_2_. Demographically heterogeneous communities were selected because they would be more likely to exhibit overlapping distributions of confounding risk factors and would allow adjustments for confounding in the analysis. Replication of exposure profiles was employed to improve the chance of including demographically comparable communities and to allow estimation of residual variance within pollution profiles. Additional details have been described previously (Berhane et al. 2004; Peters et al. 1999a, 1999b). This design resulted in contrasting exposure profiles for O_3_ and a package of correlated pollutants (PM_10_, PM_2.5_, and NO_2_) primarily of mobile source origin. This approach can be extended to other pollutants, such as ultrafine particles whose concentrations may also vary on a localized scale of ≤50 m. Selecting subjects within communities based on the distance between the home and the nearest busy roadway or other traffic density metric may maximize the exposure contrasts of ultrafines within the profiles of other pollutants such as O_3_.

Other potential valuable exposure sampling designs might consider “matrix” sampling approaches, which would draw on subsets of subjects for specific substudies or specialty projects. In the larger perspective however, maximizing differences in community exposure profiles can provide a rich population base from which to develop and inform multiple studies seeking to optimize the National Children’s Study effort.

### Exposure metrics.

Because of the large size, long duration, and diversity of outcomes and exposures of interest in the proposed National Children’s Study, the exposure assessment effort should rely on modeling to provide estimates for the entire cohort, supported by subject-derived questionnaire data. Necessary survey information on temporal–spatial–physical patterns of activity and household characteristics can be collected for the entire cohort, and targeted exposure substudies can be performed in selected subsamples of study subjects.

Issues to consider include modeling for large-scale investigations over long periods (e.g., the National Children’s Study), which is currently the only feasible approach for assigning exposure estimates for the entire cohort. This is especially true for ambient air pollutants that display significant spatial variation on urban, neighborhood, or household spatial scales.

A variety of exposure assessment modeling approaches are available, including proximity-based, geostatistical, land-use regression (LUR), dispersion, integrated meteorologic emission, and hybrid approaches involving personal sampling in combination with one or more of the above methods (Jerrett et al. 2004). Each model varies by data input requirements, software/hardware, technical expertise, and resulting accuracy and extrapolation potential.

Modeled estimates can be refined using targeted substudies designed to measure levels at geographic locations over time on the scale of spatial and temporal variation of the pollutants under study. The time resolution of the exposure estimate needs to be appropriately matched to outcomes to capture effects of frequency, magnitude, and duration of peak or episodic exposure events that may have effects during windows of vulnerability. Long-term average exposures, including average peak levels or hours above threshold levels, are likely more important for relationships with chronic disease, but this assumption needs to be evaluated for specific agents and outcomes of focus in the National Children’s Study.

Data availability and quality for model input are critically important. Central-site monitoring data can be used to assign exposure for outdoor environments, but the utility of this assignment will depend on the relative variability of the pollutant across the sampling area of interest (intra- vs. intercommunity variability issues). Estimates of indoor concentrations require individual information on home operating conditions, home source profiles and activity, factors influencing the penetration of outdoor pollutants and/or the dilution of pollutants of indoor origin (LaRosa et al. 2002; Navidi et al. 1999). Information about temporal, spatial, and physical activity patterns are also important but are likely to have insufficient time resolution over the period of study interest. Broader categories of “usual” patterns of activity, household operation, and susceptibility factors can be considered as modifying factors for the exposure–response relationship using available central-site monitoring data (Gauderman et al. 2000; Janssen et al. 2002).

An existing national system of central site monitors collects continuous data on criteria air pollutants and more limited data on hazardous air pollutants [U.S. Environmental Protection Agency (EPA) 2004]. It is possible to add additional instruments to monitoring sites to measure additional pollutants or speciate PM at reasonable cost. However, the use of central-site monitoring data for epidemiology studies requires a quality assurance activity beyond that which is used for regulatory activities as well as methods to address missing data issues. The Health Effects Institute recently funded a study to compile existing estimates of air toxics into a coherent national database. When available, these data may contribute to the National Children’s Study, and selection of the sampling sites for the National Children’s Study should take into account the location of existing and upcoming monitoring data. No similar monitoring network exists to assess exposure from indoor sources, which may need to rely on questionnaire information and substudies across regions.

Modeling of pollutants with large intra-community variation requires additional community measurements. Substudies can be designed to exploit obtainable information for modeling study subject exposures (Jerrett et al. 2005). These additional microenvironmental measurements can be used for fitting models to better estimate exposure, for model validation, and for assessment of errors in exposure assignments. Calibration studies using repeated personal monitoring may be designed and conducted to validate the exposure estimates and correct for exposure error in the analysis (Berhane et al. 2004; Fraser and Stram 2001; Mallick et al. 2002; Stram et al. 1995).

An illustration of these approaches may be seen in the USC CHS. The USC CHS framework employed a hierarchical approach for estimating exposure, ranging from the coarsest spatial estimates based on community pollutant levels measured at a single central monitor per community, to the finest spatial-scale estimates based on integrated models for individual exposure assessment. The framework involved the following pollutant measurement and modeling levels: *a*) continuous monitoring of O_3_, NO_2_, and PM_10_, and of PM_2.5_ mass and composition on a time-integrated 14-day basis, at a central monitoring station in each community; *b*) measurement of selected pollutants at multiple locations within each community; and *c*) adjustment of the central site monitor to the levels around children’s homes and schools based on a limited number of field measurements. This framework is augmented by *a*) modeling of vehicle emissions using geostatistical methods and spatial dispersion models, *b*) estimating outdoor pollutant concentrations at schools and homes for the entire study population using spatial statistical models in a hybrid microenvironmental approach, and *c*) modeling individual exposure estimates for the entire study population using unified modeling methods that integrated information with different spatial and temporal resolutions. These unified methods include community monitored pollutant levels, studies of indoor and outdoor levels in homes and schools; step counters; questionnaire-based data on time–activity patterns including commuting patterns, traffic patterns, and housing characteristics; and appropriate accounting of uncertainty in the exposure estimates.

The USC CHS developed a microenvironmental exposure model that, in principle, can provide estimates of exposures to pollutants of ambient origin in five microenvironments. These include residential outdoors, residential indoors, school outdoors, school indoors, and inside vehicles. The exposure model uses individual-level time–activity and housing survey data, residence and school-level traffic model estimates, and community-level air quality measurement data and regional transport factors to estimate short-term and long-term individual exposures. The model estimates show the largest amount of within-community variations in individual exposures of any of the models; however, validating these types of models is difficult and resource intensive (Peters 1997).

Newer modeling strategies such as LUR models are promising. LUR employs the pollutant of interest as the dependent variable and proximate land use, traffic, and physical environmental variables as independent predictors. The methodology seeks to predict pollution concentrations at a given site based on surrounding land use and traffic characteristics. The incorporation of land use variables into the interpolation algorithm detects small-area variations in air pollution more effectively than do standard methods of interpolation (i.e., kriging) (Briggs et al. 1997, 2000; Lebret et al. 2000). These methods are promising for the National Children’s Study because they can be extrapolated, based on land use coverage, without need for extensive monitoring in each location. Most major urban centers maintain land use information, and the U.S. Census has much of the information needed on population density and employment structures. The National Children’s Study could support the monitoring needed to calibrate LUR models that are regionally representative of broad land use and emission patterns. Derived coefficients could then be applied to other places within the region without need for extensive monitoring.

### Use of limited substudies for exposure refinement.

Assessment of some exposures of interest will require individual measurements of exposures using snapshots of personal and microenvironmental exposures over short periods and/or in selected microenvironments.

Issues to consider include the large number of interrelated factors that are important in designing exposure substudies. These include the substudy’s purpose, the population sample to include, whether personal or microenvironmental samples should be collected, the respondent burden, study feasibility, sample collection and analytic costs, temporal variation of exposure, subject activity patterns, household operation by residents, and uses in model validation and calibration.

These elements are nicely illustrated in the Columbia Pregnancy Cohort Study (PCS), a study performed by the Columbia University Center for Children’s Environmental Health, which has focused on the effects of pre- and postnatal exposures to air pollution on birth outcomes and neurodevelopmental and respiratory health outcomes in childhood via through recruitment and follow-up of pregnant women and their offspring (Miller et al. 2001; Perera et al. 2003, 2004a; Tonne et al. 2004; Whyatt et al. 2003). In the Columbia PCS, direct air pollution exposure assessment begins in the third trimester of pregnancy with collection of a 48-hr personal sample of PM_2.5_ and vapors for each pregnant woman. These samples are analyzed for polycyclic aromatic hydrocarbon (PAH) and pesticide concentrations (i.e., a “snapshot” measurement representing “usual” exposure). In a validation substudy, the investigators also collected sequential 2-week integrated indoor samples, analyzed for the same variables as above, for the entire third trimester (preferred over the personal snapshot as an exposure surrogate of third-trimester exposures, but obviously more intensive laborwise, cost-wise, and subjectwise). A home dust sample was also collected during the third trimester from subjects and analyzed for standard allergens relevant to maternal exposures and possible prenatal sensitization, based on evidence emerging from the Columbia PCS (Miller et al. 2001).

Another time interval of study exposure interest was the first 2 years of life, when infants/toddlers spend substantial amounts of time in the home; this may be a critical exposure window for development of allergy and asthma. Columbia PCS homes were visited when the child reached 1 year of age, and a dust sample was collected for allergen analysis. Additional sampling was performed in a subset of 25% of the homes, where 2-week samples of indoor and outdoor air PM_2.5_, black carbon, and NO_2_ were collected. These samples are being used to develop and test a spatial LUR model that will then be used to estimate exposures in the full cohort that are representative of those occurring in early childhood.

As a part of its investigations of childhood asthma in Baltimore, Maryland, the Johns Hopkins Center for Asthma in the Urban Environment (JHU Center) has conducted an intervention trial and a cohort study of asthma morbidity (Breysse et al. 2005; Swartz et al. 2004). The exposure assessment efforts for these studies include indoor and outdoor air pollution as well as indoor allergens in approximately 400 homes. The major focus of these studies was indoor air where investigators assessed 3-day average indoor PM_10_, PM_2.5_, NO_2_, O_3_, and nicotine at 3-month intervals (Breysse et al. 2005). In addition, 3-day time resolved PM was assessed using a data-logging nephalometer. Ambient PM air pollution was assessed using a monitoring site centrally located to the study area.

Results from these studies demonstrate the importance of assessing indoor air. Children, particularly young children, spend the great majority of their time in the home. Others have noted (Wallace et al. 2004) that indoor PM concentrations are generally higher than outdoor levels, and cigarette smoking as well as other household activities are responsible for this increase. In some cases, the PM contribution from cigarette smoking to indoor PM is greater than that penetrating from outdoor air. The JHU Center results indicate, for example, that a single cigarette contributes between 1 and 2 μg/m^3^ to indoor PM. In addition, a strategy that uses repeat measures allows larger time frame variability to be assessed (e.g., seasonal).

Results from the Michigan Center for the Environment and Children’s Health demonstrate the importance of focusing on the home as an important microenvironment for children’s exposure (Keeler et al. 2002; Yip et al. 2004). An important lesson from these studies is that home-based exposure assessments are feasible for studies involving hundreds of children and need to be considered in the National Children’s Study. This conclusion is particularly true for newborn children who spend essentially all of their time in the home. The microenvironments of importance include the indoor environment in a range of housing types, because there is a growing recognition that housing quality is an important predictor of indoor air pollution and can affect outdoor pollution penetration rates as well as being a general risk factor for poor health (Kingsley 2003).

As described above, the USC CHS experience suggests that exposure assignment accuracy can be improved by conducting substudies with a limited number of measurements extended temporally and spatially. In evaluating the minimal sampling needed to successfully predict long-term exposures in study communities, USC CHS investigators found that the intraclass correlation between estimated annual average of pollutants, based on 2-week subset measurements, and the true annual average was greater than 0.9 for O_3_, NO_2_, and nitric oxide in southern California, if two winter, two summer, and one spring sample were obtained. Greater numbers of samples did not appreciably improve the correlation. These results indicate that accurate estimates of the pollutant annual average levels can be obtained at homes, schools, and other central site locations with a limited number of samples. Local measurements can then be combined with concurrent central site measurements to estimate neighborhood and household scale concentrations for the entire cohort. Although the optimum number of samples may differ by region of the country or in different neighborhoods within communities, depending on the pollutants of interest and geographic and temporal variation in the processes driving air pollution, this general strategy may be of use in planning efficient National Children’s Study substudies.

### Analytic and interpretation issues.

Understanding issues of spatial/temporal correlations of air pollutants, the surrogacy of specific pollutants for components of the complex mixture, and the exposure misclassification inherent in exposure estimates will be critical in analyzing and interpreting National Children’s Study findings.

Issues to consider include the fact that air pollutants occur as complex mixtures of gases and particles, but coexisting constituents may covary, based on their common sources or photochemical pathways. The ambient level of one pollutant may therefore be a surrogate for other pollutants arising from the same source, so interpretation of findings for individual pollutants must account for this surrogacy (Manchester-Neesvig et al. 2003; Sarnat et al. 2001). Identification of pollutant sources therefore provides a potentially important mechanism to evaluate source-specific health effects and can ultimately lead to effective strategies for reducing population exposure.

Substudies among subjects in differing geographic locations may be useful for defining pollutant relationships. For example, in assessing PM, chemical tracers have been identified that can serve as “fingerprints” for individual sources, or source types, of air pollution (Laden et al. 2000; Manchester-Neesvig et al. 2003; Sarnat et al. 2002). This type of information can be used to apportion contributions to the measured PM mass on a per sample basis, along with providing data critical to the assessment and interpretation of health effects associated with individual chemical components of PM. Quantitative assessments of source contributions for large data sets are often determined using a statistical receptor modeling approach. This type of data analysis is best suited to longitudinal study designs and can be limiting because it may require collection of a large number of samples to obtain robust results.

The recent successful development and deployment of several types of continuous portable PM mass and number monitors offer the potential for producing real-time (< 5-min interval) data. The continuous data collection format of these samplers allows a better understanding of source emission patterns and exposures, especially in urban environments, and can be used to enhance investigations of short-term peak exposures. These highly time-resolved exposure data can be coupled with personal time–activity pattern data to quantitatively identify exposures from specific emission sources. To date, real-time PM samplers do not yet offer the ability to determine PM chemical speciation. A combination of methodologic approaches (employing chemical tracers and continuous PM number and mass count information) may improve the ability to identify specific sources and source types contributing to the measured exposure to PM and other pollutants.

Exposure misclassification is a critical issue for exposure assessment efforts, especially modeled exposures. In most large cohort studies, it is not possible to accurately measure the true personal exposure of individuals over the time interval that is most relevant for the outcomes of interest. Thus, virtually all exposure assessments provide at best estimates of true exposures, with some error. Errors may arise because of temporal factors (e.g., the exposure metric captures only a snapshot of the relevant time interval) or spatial factors (e.g., the exposure metric is collected at a location different from where the subject lives and breathes). Additionally, inherent imprecision in the specific method selected for study application may also result in some measurement error. For the results of the study to ultimately be interpretable, it is important in designing the study for investigators to analyze the nature of the exposure misclassification errors that are likely to be present. Quantitative estimates of exposure errors can be obtained by carrying out calibration substudies where results from more complete exposure metrics are compared with results from the modeled metrics (Berhane et al. 2004; Fraser and Stram 2001; Mallick et al. 2002; Sarnat et al. 2001; Stram et al. 1995). Bayesian statistical frameworks may assist with assessing the impact of measurement error on the exposure–response relationships (Berhane et al. 2004).

### Modifiers of exposure–outcome relationships.

“Usual” temporal, spatial, and physical patterns of activity can be used as modifiers of the exposure–outcome relationships. Highly time-resolved activity information over the study period of interest may not be necessary, and is not likely to be available, for all National Children’s Study participants throughout the study. Personal exposure estimates, based on time in microenvironments, are likely to be associated with large uncertainties. “Usual” patterns of activity, such as time usually spent outdoors, can be collected by questionnaire and used as modifiers of exposure–outcome relationships (Gauderman et al. 2002). Activity-level assignments may be important in moving from exposure to delivered dose of an airborne pollutant to the lung. For example, for asthma prevalence and incidence, USC CHS investigators saw little association with community levels of exposure. However, when physical activity was considered, O_3_ was strongly associated with asthma incidence (where variation entered from increased ventilation rates associated with exercise and likely increased dose to the lung). An important challenge for the National Children’s Study is assessing activity patterns among mothers, infants, and young children.

For extremely large study populations for which individual questionnaires may be impractical to administer and/or collect, randomized sampling schemes or oversampling in certain nested subsamples of possible increased interest may be worth careful consideration.

### Use of biomarkers.

Biomarkers of exposure offer utility for evaluation of specific exposures that have multiple routes of exposure. For specific airborne pollutants, exposure assessments may need to consider multiple routes of human exposure. In addition to inhalation, dermal absorption and oral ingestion may be important pathways of exposure for pollutants of interest with regard to young children, infants, and pregnant or lactating mothers. The use of exposure biomarkers is one potentially valuable approach in this area (Weaver et al. 1998). Interpreting the relationship between these markers and exposures, however, is a complex function of the timing and routes of exposure, and of the pollutant toxicokinetics. As discussed above, temporal–spatial–physical patterns of activity will almost surely affect this dynamic in important ways, from modification of ventilation rates to facilitated dermal absorption during periods of elevated, increased, or extended activities. As exposure assessment tools, biomarkers offer the potential advantage of integrating the net effect of all of these factors in producing a given internal dose for a given individual. Such measurements may better represent true health-relevant exposures for an individual than any external measure of exposure can.

Biomarker measurements are substantially integrated into the exposure and health assessment designs of the Columbia PCS. From an exposure perspective, biomarkers focus on DNA-bound PAHs (Perera et al. 2004a, 2004b), pesticides in blood plasma and meconium (Perera et al. 2003; Whyatt et al. 2001, 2003, 2004), and the environmental tobacco smoke (ETS) metabolite cotinine in urine (Perera et al. 2004b), beginning with maternal and infant cord blood samples at birth, and continuing with follow-up assessments in the child at 2 and 5 years of age. PAH-DNA adducts also can be viewed as early measures of procarcinogenic health effects (Perera et al. 2004b). Other effect-related biomarkers focus on the time course of sensitization to environmental allergens, including measurements of maternal, cord-blood, and child IgE, and production of proinflammatory cytokines or proliferation of mononuclear cells in response to specific allergens (Miller et al. 2001).

The integration of newly developed pesticide biomarkers within the epidemiologic design of the Columbia PCS has made possible significant new advances in our understanding of the health effects and patterns of exposures to pesticides among urban women and children (Perera et al. 2003; Whyatt et al. 2001, 2003, 2004). A wide range of pesticides have been shown to be quantifiable in the plasma of women and their newborns, with significant correlations between maternal and cord blood levels in many cases (Whyatt et al. 2003). For some but not all pesticides, correlations also were demonstrated between plasma levels at birth (either cord blood or maternal) and air measurements collected during the third trimester of pregnancy. Cord plasma, but not air, levels of the insecticide chlorpyrifos and diazinon were significantly associated with decreased birth weight and length (Whyatt et al. 2004). Of particular significance, levels of several pesticides in both air and plasma showed significant declines across women enrolled before and after the U.S. EPA insecticide phase-out (Whyatt et al. 2003). Furthermore, associations with adverse birth outcomes were significant only for infants born before the phase-out (Whyatt et al. 2004). These findings illustrate the utility of well-targeted biomarker measurements, in conjunction with health and external exposure measures, for birth cohort studies.

Cotinine and nicotine as markers for ETS, an important source of PM exposure, has a long history of use in biomonitoring. Hair nicotine has the potential to provide estimates of ETS exposure over a 2–3 month period or longer (Jaakkola and Jaakkola 1997), and other nicotine metabolites (e.g. cotinine) may be useful indicators of both exposure and bioavailability.

## Summary

The National Children’s Study offers a unique opportunity to understand the adverse effects of air pollution on a broad range of interrelated outcomes during the critical period of early life development and growth. Six recommendations for air pollution exposure assessment are proposed from lessons learned in the Children’s Centers.

- **National Children’s Study subject selection.** Study populations should be selected to maximize spatial-scale exposure contrasts for the pollutants of interest. Because multiple pollutants are of interest for the National Children’s Study, priorities must be established to allow identification of individuals with a wide range of exposure profiles for those key pollutants of study interest.
- **Exposure metrics**. Because of the large size, long duration, and diversity of outcomes and exposures of interest in the proposed National Children’s Study, the exposure assessment effort should rely on modeling to provide estimates for the entire cohort, supported by subject-derived questionnaire data. Necessary survey information on temporal–spatial–physical patterns of activity and household characteristics can be collected for the entire cohort, and targeted exposure substudies can be performed in a selected subsample of study subjects.
- **Use of limited substudies for exposure refinement.** Assessment of some exposures of interest will require individual measurements of exposures using snapshots of personal and microenvironmental exposures over short periods and/or in selected micro-environments.
- **Analytic and interpretation issues.** Understanding issues of spatial–temporal correlations of air pollutants, the surrogacy of specific pollutants for components of the complex mixture, and the exposure misclassification inherent in exposure estimates will be critical in analyzing and interpreting findings from the National Children’s Study.
- **Modifiers of exposure–outcome relationships.** “Usual” temporal, spatial, and physical patterns of activity can be used as modifiers of the exposure/outcome relationships.
- **Use of biomarkers.** Biomarkers of exposure may be required for evaluation of specific exposures that have multiple routes of exposure.

We have learned that there are many challenges to assessing air pollution exposures to children. To overcome these challenges, the National Children’s Study will need to commit extensive resources to exposure assessment activities. With optimal subject selection, exposure estimates can be modeled for the entire cohort, supported by direct measurement of selected pollutants in a subset of the study population. Biomonitoring is likely to be a valuable adjunct to the exposure assessment design, helping to trace the mechanistic linkages between exposures and effects. Prioritization of pollutants of study interest and developmental periods of study focus would allow optimization of the study design for the National Children’s Study to maximize contrasting pollution profiles and enhance the ability to assess exposure–response relationships.

## Figures and Tables

**Table 1:** Centers for Children’s Environmental Health and Disease Prevention Research air pollution exposure assessment experience relevant to the National Children’s Study.

	Columbia University	Johns Hopkins University	University of Michigan	USC Children’s Health Study	University of Southern California
Sample population	500 pregnant women enrolled in the third trimester, and children followed from birth through age 5	~ 250 children with asthma in urban Baltimore (ages 2–12)	300 children, moderate to severe asthma, 7–11 years of age at baseline	~ 6,000 public school children, 9–18 years of age in four specific age cohorts, from 12 southern California communities	202 Los Angeles public school children, 6–16 years of age with asthma and allergy to house dust mite or cockroach
Outcome(s)	Asthma and neurodevelopment; follow-up at multiple time points starting at birth; outcome metrics include questionnaires, biomarkers, clinical assessments, neurobehavioral assessments	Asthma severity	Daily symptom diaries and pulmonary function (PEF, FEV_1_)	Pulmonary function (PFTs), symptoms (from annual medical and residential histories for 10 years), school-reported absences, food-frequency dietary information, physical activity, smoking and ETS, GxE interactions	Asthma severity
Study design	Prospective birth cohort study with exposures and outcomes measured at multiple time points starting during the third trimester of pregnancy	Longitudinal intervention trial (*n* = 100); longitudinal cohort study (*n* = 150); cross-sectional case–control study	Longitudinal intervention trial	Cross-sectional survey (*n* ~ 3,600); longitudinal cohort study (*n* ~ 5,600)	Randomized trial of allergen-reduction strategies
Agents assessed	Personal PAH and pesticide exposures of mother in third trimester; dust allergens prenatal, 12 months, 36 months, and 60 months; indoor/outdoor PM_2.5_, black carbon, and NO_2_ at 12 months in subset; biomarkers for ETS, PAH–DNA adducts, pesticides	Indoor/outdoor air pollutants (PM_10_, PM_2.5_, O_3_, nicotine); airborne endotoxin and mouse allergen; allergens in reservoir dust (cockroach, mouse, dust mite, cat, dog)	Personal/indoor/outdoor air pollutants (PM_10_, PM_2.5_, O_3_, nicotine); PM components (trace elements, EC, OC, endotoxin)	Outdoor air pollutants [O_3_, NO_2_, PM_10_, PM_2.5_, acid vapor (HNO_3_, formic, acetic) EC, OC, PM speciation (SO_4_, NO_3_, NH_4_, CI)], PAHs, endotoxin, air toxics, ETS, cigarette smoke	Settled allergens (dust mite and cockroach) and endotoxin; cockroach counts
Other exposure determinants	GIS assessment of traffic proximity; social condition and stress; home characteristics	Home inspection, time– activity data, GIS location, meteorology	Home inspection, time– activity data, GIS location, meteorology	Annual residential history by written survey; time–activity data, GIS location, traffic density, and proximity	Housing characteristics and condition, reported and observed behavior, humidity and moisture
Assessment strategy	Prenatal exposures to PAH based on personal sampling and cord blood PAH–DNA adducts at birth; allergen exposures based on dust measures; postnatal air pollution exposures based on prediction model developed in subset	Primary exposure assignment based on indoor air pollutants, and allergens; secondary exposure assignment using microenvironmental model with indoor/outdoor air pollution combined with time–activity information	Primary exposure assignment using personal/indoor/outdoor air pollutants; secondary exposure assignment using microenvironmental model	Primary exposure assignment based on community ambient monitors; secondary exposure assignment using microenvironmental model with outdoor air pollution combined with home characteristics and time– activity information	Assessment of only indoor settled dust; no outdoor assessment

Abbreviations: CI, chlorine; EC, elemental carbon; FEV_1_, forced expiratory volume in 1 sec; GIS, geographic information system; GxE, gene–environment interaction; OC, organic carbon; PEF, peak expiratory flow; PFT, pulmonary function test.

**Table 2:** Spatial scales of variability for ambient air pollutants.

Compound	Regional scale (100–1,000 km)	Urban scale (4–50 km)	Neighborhood scale (50 m to 4 km)	Household scale (≤50 m) outdoors and indoor
Primary PM_2.5_ constituents				
EC from combustion		x	x	x
Organics, including PAHs		x	x	
Metals, including chromium VI, cadmium, lead, beryllium, nickel, arsenic, iron, manganese		x	x	x
Other constituents from road dust, wood smoke, construction dust, and industrial sources		x	x	
Secondary PM_2.5_ constituents				
Sulfate	x			
Nitrate	x	x		
Ammonium	x	x		
Secondary organics	x	x		
Primary PM_2.5–10_ constituents				
Organics, including PAHs		x	x	x
Metals, including chromium VI, cadmium, lead, beryllium, nickel, arsenic, iron, manganese		x	x	
Other constituents from road dust, wood smoke, construction dust, and industrial sources		x	x	
Primary PM _> 10_ constituents				
Pollen grains			x	x
O_3_	x	x		
Nitric oxide		x	x	
NO_2_		x	x	
Sulfur dioxide		x	x	
Carbon monoxide			x	x
Volatile organic compounds				
Benzene		x		x
1,3-Butadiene		x		x
Formaldehyde		x		x
Acetaldehyde		x		x
Acrolein		x		x
Vinyl chloride		x		x
Carbon tetrachloride		x		x
Chloroform		x		x
Propylene dichloride		x		x
Methyl chloride		x		x
Trichloroethylene		x		x
Tetrachloroethylene		x		x
Naphthalene		x		x
Mercury	x	x		

Bioaerosols, including endotoxin, house dust allergens, fungal spores, and pollen grains, also vary considerably on the household and neighborhood scales; however, they were not included in this analysis.
